# Igf1 and Pacap rescue cerebellar granule neurons from apoptosis via a common transcriptional program

**DOI:** 10.1038/cddiscovery.2015.29

**Published:** 2015-09-07

**Authors:** B Maino, V D’Agata, C Severini, MT Ciotti, P Calissano, A Copani, Y-C Chang, C DeLisi, S Cavallaro

**Affiliations:** 1 Institute of Neurological Sciences, Italian National Research Council, 95126 Catania, Italy; 2 Department of Biomedical and Biotechnological Sciences, Section of Human Anatomy and Histology, University of Catania, 95123 Catania, Italy; 3 Institute of Neurobiology and Molecular Medicine, Italian National Research Council, 00143 Roma, Italy; 4 European Brain Research Institute, 00143 Roma, Italy; 5 Department of Drug Sciences, University of Catania, 95125 Catania, Italy; 6 Center for Advanced Genomic Technology, Boston University, Boston, MA 02215, USA; 7 Department of Biomedical Engineering, Boston University, Boston, MA 02215, USA

## Abstract

A shift of the delicate balance between apoptosis and survival-inducing signals determines the fate of neurons during the development of the central nervous system and its homeostasis throughout adulthood. Both pathways, promoting or protecting from apoptosis, trigger a transcriptional program. We conducted whole-genome expression profiling to decipher the transcriptional regulatory elements controlling the apoptotic/survival switch in cerebellar granule neurons following the induction of apoptosis by serum and potassium deprivation or their rescue by either insulin-like growth factor-1 (Igf1) or pituitary adenylyl cyclase-activating polypeptide (Pacap). Although depending on different upstream signaling pathways, the survival effects of Igf1 and Pacap converged into common transcriptional cascades, thus suggesting the existence of a general transcriptional program underlying neuronal survival.

## Introduction

Neuronal apoptosis represents an intrinsic suicide program by which a neuron orchestrates its own destruction. It is characterized by specific morphological and biochemical events, including fragmentation of nuclear DNA, breakdown of the cellular cytoskeleton and the bulging out of the plasma membrane (blebbing), which may lead to the detachment of the so-called apoptotic bodies.^1^ During normal nervous system development, physiologically appropriate neuronal loss contributes to a sculpting process that removes approximately one-half of all neurons born during neurogenesis.^2^ Neuronal loss subsequent to this developmental window is physiologically inappropriate for most systems and can contribute to neurological deficits, for example, neurodegenerative diseases such as Alzheimer's and Parkinson disease.^3^ Hence, elucidating the molecular mechanisms underlying neuronal apoptosis may contribute to understanding the basis of developmental biology and human neuropathology.

Cerebellar granule neurons (CGNs) are the most abundant neuronal cell type in the mammalian brain and are used as a model, either *in vivo* or *in vitro*, to study neuronal apoptosis.^4,5^ Loss of neurotrophic supply and/or activity-dependent survival signals can induce apoptosis in CGNs. The relative contribution of these components correlates to the neuronal type and age. During early stages of postnatal development, it is assumed that apoptosis of granule cells reflects the failure of these neurons to obtain adequate amounts of specific neurotrophic factors.^4^ In the adult, mossy-fiber axotomy is followed by apoptosis of CGNs, highlighting the crucial role of afferent inputs on the survival of these cells.

Primary cultures of CGNs have been extensively utilized to examine the signal transduction mechanisms underlying neuronal apoptosis. In this *in vitro* paradigm, CGNs undergo rapid apoptotic cell death within 24 h after the removal of serum and lowering of extracellular potassium from 25 to 5 mM.^5^ The apoptotic process requires protein transcription and synthesis, becoming irreversible after the first 6 h following its induction.^6^ Before this ‘commitment point’, CGNs can be rescued by the activation of specific signal transduction pathways or by the treatment with specific neurotrophic factors. In our previous studies, we identified two important growth factors capable of preventing apoptosis of CGNs: insulin-like growth factor-1 (Igf1)^5^ and pituitary adenylyl cyclase-activating polypeptide (Pacap).^7^ The survival effects of these growth factors are mediated by different receptors and intracellular second messengers.^5,7^ Although these signaling pathways converge into the nucleus and regulate gene expression, the transcriptional program underlying neuronal survival is still unknown. In the present work, we carried out whole-genome expression profiling to investigate the rescue effects of Igf1 and Pacap in CGNs and identified crucial genes and pathways at the intersection of neuronal apoptosis and survival.

## Results

### Induction of apoptosis and rescue by Igf1 and Pacap

**Figure 1:**
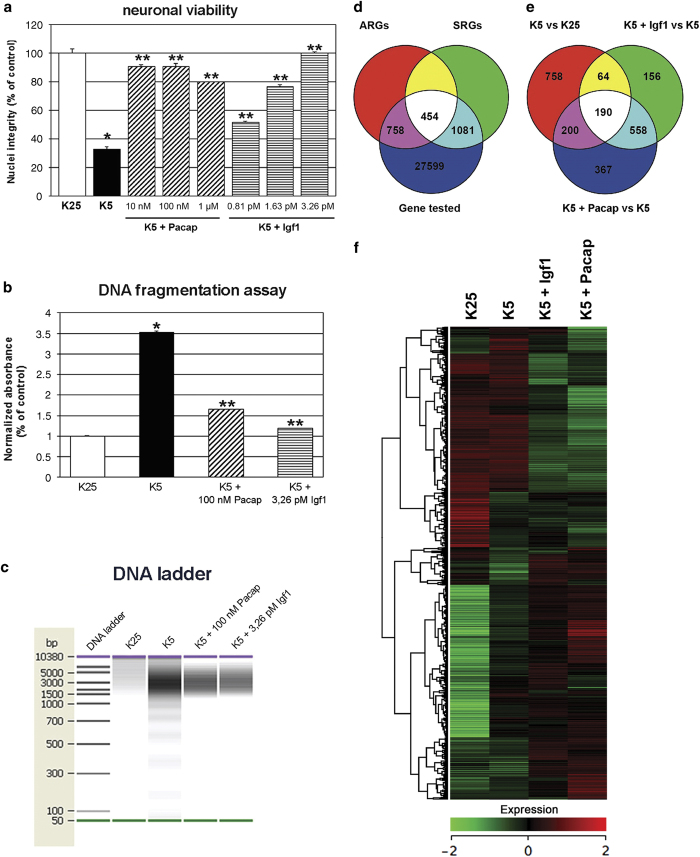
Induction of apoptosis in CGNs and rescue by Igf1 and Pacap treatment. (**a**) Cultured CGNs at 6 days ‘*in vitro*’ were switched into serum-free medium containing lower concentrations of extracellular K+ (5 instead of 25 mm) for an acute induction of apoptotic death. Forty-eight hours later, neuronal viability was assessed by counting the number of intact nuclei. Values for neuronal viability represents the mean±S.E.M. of four to eight determinations in two different experiments. Determination of oligonucleosomes generated by cleavage of nuclear DNA was performed with ELISA (**b**) and electrophoresis on a microchip device (**c**). Values for DNA fragmentation assay represent the mean±S.E.M. of four to eight determinations in two different experiments. *P*<0.001 *versus* K25 (*) or K5 (**) were determined by one-way ANOVA followed by Tukey *post hoc* test (**a** and **b**). (**d** and **e**) Genes differentially expressed in apoptotic CGNs (K5 *versus* K25) were defined as ‘Apoptotic related genes’. Genes differentially expressed in rescued CGNs (K5+Igf1 *versus* K5; K5+Pacap *versus* K5) were defined as ‘Survival related genes’. (**f**) Hierarchical cluster of genes related to apoptosis and rescue of CGNs. ARGs and SRGs were arranged in a dendrogram in which the pattern and length of the branches reflect the relatedness of the expression levels under four different experimental conditions. Green, black and red cells, respectively, are transcript levels below, equal or above the median abundance across all conditions. Color intensity reflects the magnitude of the deviation from the median. The Pearson correlation coefficient of SRG fold ratios (K5+Igf1/K5, K5+Pacap/K5) is 0.97.

CGNs undergo apoptotic cell death after the removal of serum and lowering of extracellular potassium from 25 to 5 mM,^5^ and can be rescued by Igf1^5^ and Pacap treatments.^7^ To confirm this in our *in vitro* paradigm and select the doses of Igf1 and Pacap having similar efficacy, we used three diverse methods to assess apoptosis and survival. Neuronal viability was assessed by counting the number of intact nuclei, whereas determination of oligonucleosomes generated by cleavage of nuclear DNA was performed with enzyme-linked immunosorbent assay (ELISA) and electrophoresis on a microchip device (Figures 1a–c). Forty-eight hours after the induction of apoptosis, neuronal viability of CGNs was ~32% of control and DNA fragmentation increased 3.5-folds (Figures 1a and b).

Treatment with Igf1 or Pacap prevented most of CGNs from undergoing apoptosis and the maximal efficacy reached by 3.26 pm Igf1 was similar to that obtained with 100 nm Pacap (Figures 1a–c).

### Whole-genome expression changes underlying apoptosis and survival

By using oligonucleotide microarrays, we monitored whole-genome expression profiles of CGNs after induction of apoptosis and following rescue by a maximal effective dose of Igf1 (3.26 pm) or Pacap (100 nm). To exploit the comprehensiveness of our data, we investigated changes on the level of individual genes and in functional gene groups.

### Identification of differentially expressed genes

When gene expression profiles in control CGNs (K25) were compared with those of CGNs 6 h after induction of apoptosis (K5), 1212 genes, operationally defined as ‘Apoptotic Related Genes’ (ARGs), showed significant changes of gene expression (Figures 1d and e). By comparing gene expression profiles in CGNs 6 h after induction of apoptosis (K5) with those of apoptosis-rescued CGNs by treatment with Igf1 and Pacap, 1535 genes were found to be differentially expressed and were operationally defined as ‘Survival Related Genes’ (SRGs; Figures 1d and e). Among these, 968 and 1315 genes were differentially expressed after treatment with Igf1 and Pacap, respectively (Figure 1e).

Although the survival effects of Igf1 and Pacap are initiated by different receptors and activate a variety of intracellular second messengers,^5,7^ they were propagated by common transcriptional cascades and showed striking similarities. Indeed, 748 out of 1535 SRGs overcoming a stringent cutoff level were common to Igf1 and Pacap (Figure 1e). The Pearson correlation coefficient between Igf1 and Pacap for all 1535 SRGs was 0.97, further supporting the similarity of their genomic effects (Figure 1f).

A comprehensive picture of transcriptional changes associated with apoptosis and survival is shown in Figure 1f, where ARGs and SRGs (a total of 2293 genes) are grouped on the basis of similarity in their expression patterns with a hierarchical clustering method. The large impact of apoptosis and survival on the CGNs transcriptome, together with the remarkable concordance of Igf1- and Pacap-induced effects can be distinguished by the color matrix. The hierarchical cluster shows in an unequivocal manner that gene expression patterns of Igf1 and Pacap were highly similar to each other (Figure 1f).

Although our data represent the average gene expression from four replicates, we confirmed the reliability of the microarray data by quantitatively validating the differential expression of seven genes in each of the four experimental conditions using quantitative RT-PCR (Supplementary Table S2). Using RT-PCR, the pattern of gene expression from sample to sample closely paralleled that observed using microarray. The mean±S.D. of the correlation coefficients between the two profiles was 0.95±0.06.

### Identification of functional pathways

**Figure 2:**
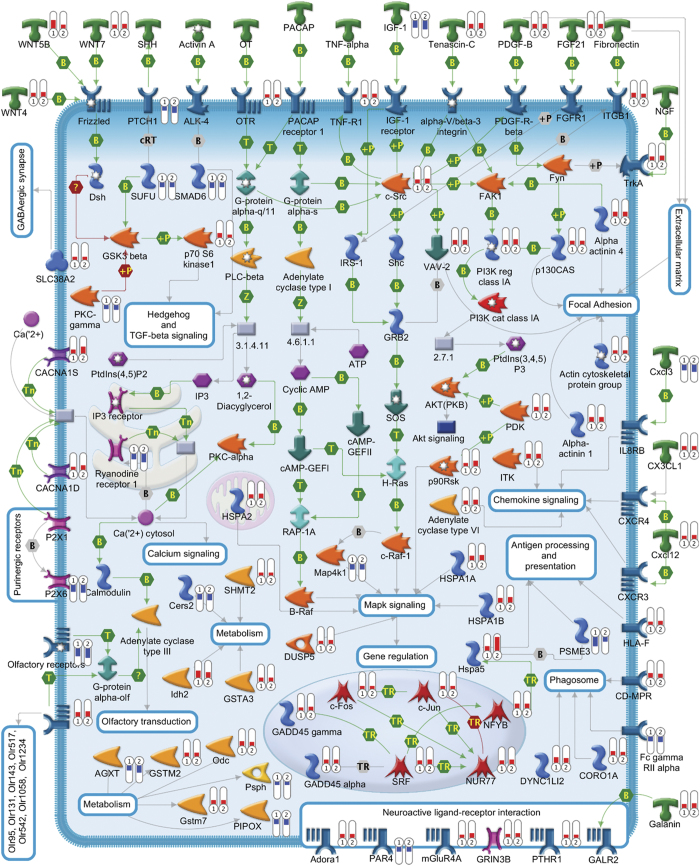
A comprehensive picture showing gene expression changes and crosstalk of pathways associated with Igf1 and Pacap rescue of apoptotic CGNs. This figure, designed using the MetaCore software (Thomson Reuters, New York, NY, USA), illustrates the gene expression profiles and the 14 signaling pathways that are involved in Igf1 and Pacap neuroprotection of apoptotic CGNs. Each encoded protein is labeled with two thermometers that indicate gene expression changes under the following experimental conditions: K5+Igf1 (first thermometer) and K5+Pacap (second thermometer). More specifically, downward thermometers have a blue color and reflect downregulated expression, whereas upward thermometers have a red color and reflect upregulated expression. Further explanation of symbols used in the pathway map are listed in Supplementary Figure S2.

To analyze gene expression changes in the context of known biological pathways, we used PathWay Enrichment Analysis (PWEA)^8^ and identified 14 KEGG pathways that were deregulated following both Igf1 and Pacap treatments. These pathways can be organized in three main functional categories: cellular processes (focal adhesion; phagosome; antigen processing and presentation), metabolism (glycine, serine and threonine metabolism; glutathione metabolism; sfingolipid metabolism) and signal transduction (chemokine signaling; neuroactive ligand–receptor interaction; olfactory transduction; calcium signaling; GABAergic synapse; hedgehog signaling; TGF-beta signaling; MAPK signaling). All differently expressed pathways are shown in Supplementary Materials (Supplementary Figures S1A and S1M). Genes with significant deregulated changes in all these pathways are summarized in figure 2.

## Discussion

In recent years, the advent of full-genome sequencing and high-throughput technologies has offered a new approach to decode the mechanisms underlying neuronal apoptosis and survival, unraveling a systems biology-based perspective. The ability of a cell to promote or evade apoptosis depends on the activity of an integrated network of genes and their encoded proteins, which never work alone but interact with each other in highly structured and incredibly complex ways, forming molecular circuits that correspond perfectly to cell functional specifications.

This study reports for the first time a whole-genome analysis of neurons induced to apoptosis and rescued by growth factors. We found striking similarities in CGNs rescued by Igf1 and Pacap, despite the fact that they act on different receptors. This raised our interest in exploring individual genes and crosstalk of transcriptional pathways that govern CGN survival. In the following paragraphs, we discuss these transcriptional changes highlighting statistically significant changes in genes and functional pathways that accompany CGN rescue from neuronal apoptosis.

### Cellular processes

Focal adhesions are important cell–matrix junctions that have an essential role in intracellular survival signaling. Our analysis revealed that the overexpression of genes encoding focal adhesion proteins during CGN rescue may favor cell survival. Their encoded proteins participate in the structural link between membrane receptors and the actin cytoskeleton (Tnc, Itgb1, Actn1, Actn4 and Actin cytoskeletal groups), whereas others are adaptors (Vav2, Bcar1, Src, Pik3r1 and Pdk1) that contribute to initiate downstream signaling events, leading to neuroprotection^9–17^ (Supplementary Figure S1A).

Growing evidence supports an active role for the dysregulated autophagy-lysosome pathway in neuronal cell death and neurodegeneration. Our microarray analysis showed that Igf1 and Pacap rescue is associated with the differential expression of autophagic regulator transcripts. Increased expressions of M6pr and Coro1a, as well as a decrease in Fcgr2a, have already been associated with anti-apoptotic effects.^18–20^ Similarly, a high level of Dync1li2 was found to have a key role in recycling endosome localization^21^ (Supplementary Figure S1B).

Differential regulation of genes encoding proteins involved in the antigen processing and presentation pathway may underlie preselection of immunologically important antigenic determinants in dying cells. Consistent with this view, we observed the differential expression of *RT1-A1*, *Hspa5*, *Nf-Yb* and *Psme3* in rescued CGNs, all of which are known to exert an anti-apoptotic effect^22–25^ (Supplementary Figure S1C).

### Metabolism

The sequence of biochemical changes occurring during amino-acid biosynthesis and metabolism in neuronal cells is fundamental in maintaining life-sustaining chemical transformations. In agreement with this is the differential expression of a number of genes encoding enzymes involved in the glycine, serine and threonine metabolism. Downregulation of Agtx, Psph, Pipox, which are mostly found in the peroxisomes, may represent an adaptive response to counteract stress-induced death signaling.^26–28^ In addition, a high level of Shmt2 has been found to confer neuroprotection^29^ (Supplementary Figure S1D).

Our analysis also revealed an overexpression of five genes encoding glutathione-dependent enzymes (Idh2, Gsta3, Gstm2, Gstm7 and Odc1) that have already been related to antioxidant and anti-apoptotic effects in various cell types, such as CGNs^30–34^ (Supplementary Figure S1D).

The sphingolipid pathway is also regulated during Igf1 and Pacap rescue and includes a significantly decreased expression of apoptotic Cers2^35^ (Supplementary Figure S1D).

### Signal transduction

A large number of genes mediating signaling cascades from the cell surface to the nucleus was involved in CGN rescue. Among these, we observed the differential expression of genes encoding chemokines, neuroactive ligands, their receptors or downstream enzymes (*Cxcl12*, *Cx3cl1*,* Cxcl3*,* Gal*,* Adora1*,* F2rl3*,* Pthr1*,* Oxtr*,* mGluR4*,* Grin3b*,* Adcy6*,* Itk *and* PKCzeta*) that are known to counteract cell death because of various harmful conditions^36–48^ (Supplementary Figures S1 E and F).

The reduction of a vast number of olfactory receptor transcripts, during apoptosis rescue, suggests that they may perform other functions than those previously reported^49^ (Supplementary Figure S1G).

Regulation of calcium homeostasis has been extensively involved in cell survival.^50^ Accordingly, we observed the overexpression of *Cacna1d*, *Cacna1s*, *Slc8a1* and the decrease of *P2rx6* and *Ryr1*, which are all known to control calcium signaling and render cells less vulnerable to a wide variety of apoptotic stimuli^51–55^ (Supplementary Figure S1H).

Our results also emphasized the critical role of GABAergic synaptic signaling during CGN rescue, as the overexpression of a sodium-dependent amino-acid transporter, Slc38a2, together with the above-described proteins (Adcy6, Cacna1d and Prkcg), is known to elicit survival responses^56^ (Supplementary Figure S1I).

The overexpression of three wnt ligands (Wnt4, Wnt5b and Wnt7a) is in line with the survival effects exerted by the Wnt family in neuronal cells.^57^ Moreover, the low expression of Ptch1 (the receptor for hedgehog) and Sufu (a common regulator of wnt and hedgehog signaling) in rescued CGNs is consistent with previous evidence obtained in different cell types^58,59^ (Supplementary Figure S1L).

A number of genes encoding TGF-beta signaling proteins are regulated by Igf1 and Pacap. Among these, high levels of Rps6kb1 and a loss of Smad6 have been demonstrated to counteract apoptosis^60^ (Supplementary Figure S1L).

One of the major molecular mechanisms for controlling cell survival is the MAPK signaling.^61^ In rescued CGNs, a number of genes encoding ligands or receptors related to the MAPK signaling (*Pdgfb*,* Fgf21*,* Ntrk1 *and *Tnfrsf1a*) have been involved in neuronal survival.^62–65^ Similarly, genes encoding enzymes, such as increased p90Rsk and Dusp5, as well as decreased Prkcg and Map4k1, have been associated with neuroprotection.^66–69^ We also observed the enhanced expression of genes encoding pro-survival heat shock proteins (*Hspa1a*, *Hspa1b* and *Hspa2*), genotoxic stress proteins (*Gadd45a* and *Gadd45g*) and transcription factors (*Nr4a1*, *Jun*, *Fos* and *Srf*)^70–77^ (Supplementary Figure S1M).

## Conclusions

Our analysis offers for the first time a genomic view of the changes underlying the rescue from apoptosis by Igf1 and Pacap treatments of rat CGNs, the most common neuronal paradigm used to examine programmed cell death by trophic deprivation. Although acting through different upstream signaling pathways, most of the survival effects of Igf1 and Pacap were propagated by common transcriptional cascades, and showed striking similarities. These unpredicted results suggest the existence of a previously unknown transcriptional program specifying neuronal survival. Although commitment to apoptosis by CGNs is known to require *de novo* gene expression^5^ and has been the purpose of previous investigations, the transcriptional program underlying apoptosis rescue has been neglected. To our knowledge, this is the first time that investigated and compared transcriptional signaling pathways induced by rescue factors showed striking similarities. Elucidating the molecular mechanisms underlying neuronal apoptosis and rescue may contribute to the understanding of the molecular basis of neurodegeneration and aid the development of new therapeutic interventions. Several proteins encoded by differentially expressed genes, at the intersection of apoptosis and survival, are targets of pharmacological compounds (Supplementary Table S1). Their exploitation may help interfering with the intracellular pathways described here and guide novel therapeutic strategies for neurodegenerative diseases.

## Materials and Methods

### Materials

All the substances were obtained from Sigma-Aldrich (Milano, Italy), unless otherwise specified.

### Neuronal cultures

Primary cultures of CGNs were prepared from 8-day-old Wistar rats (Charles River, Calco, Italy) and were cultured as previously described.^7,78^ In brief, cerebella were sliced and tissue was dissociated through trypsinization in 0.025% trypsin solution (15 min at 37 °C) and trituration in presence of DNase (0.01%) and trypsin inhibitor (0.05%). Dissociated cells were collected through centrifugation and resuspended in basal Eagle’s medium supplemented with 10% fetal calf serum, 25 mm KCl, 2 mm glutamine and 100 *μ*g/ml gentamycin. Cytosine arabinofuranoside (10 *μ*M) was added after 18 h of culture to inhibit the growth of non-neuronal cells.

### Assessment of neurotrophic activity

After 6 days ‘*in vitro*’, extracellular KCl was shifted from 25 to 5 mm for neuronal apoptotic death induction. After two washes with serum-free BME containing 5 mm KCl, neurons were incubated with the same medium up to 48 h (K5), whereas control neurons were incubated with serum-free medium supplemented with 25 mm KCl (K25). K5 neurons were also treated with human recombinant Igf1 or Pacap.

The rescue effects of Igf1 and Pacap were determined by assessing neuronal viability, oligonucleosome formation and DNA fragmentation. Neuronal viability was assessed by counting the number of intact nuclei according to the method previously described.^79^ Culture medium was removed and replaced by 0.5 ml of a detergent containing lysing solution (0.5% ethylhexadecyldimethylammonium bromide, 0.28% acetic acid, 0.5% Triton X-100, 3 mm NaCl, 2 mM MgCl_2_, in PBS pH 7.4). After a few minutes, the cells were collected and intact viable nuclei were counted in a hemocytometer. Broken or damaged nuclei were not included in the counts. Quantitative determination of cytoplasmic histone-associated DNA fragments (mono- and oligonucleosomes) was performed by a photometric enzyme immunoassay (Cell Death Detection ELISA^PLUS^, Roche Diagnostics, Monza, Italy), as suggested by the manufacturer's protocol. Analysis of DNA fragmentation was performed with the Apoptotic DNA Ladder Kit (Roche Diagnostics), as specified in the instruction manual. Fragmented DNA (250 ng) was evaluated using a DNA 7500 chip and a 2100 Bioanalyzer (Agilent Technologies) with the protocol outlined by the manufacturer.

### Microarray experiments

Microarray experiments were performed in serum-deprived cells after 6 h switch from 25 to 5 mm with or without treatments with 100 nm Pacap or 3.26 pm Igf1. Control cells were grown in serum-free medium supplemented with 25 mm KCl (K25). Total RNA was extracted with Trizol (Life Technologies, Monza, Italy) from four biological replicates for each of the experimental conditions (K25, K5, K5+Igf1, K5+Pacap). RNA integrity was confirmed by using a RNA chip and a 2100 Bioanalyzer (Agilent Technologies), with the protocol outlined by the manufacturer. Complementary RNAs (cRNAs) labeled with Cy3-CTP were synthesized from 1 *μ*g of total RNA of each sample using the Low RNA Input Fluorescent Linear Amplification Kit (Agilent Technologies), following the manufacturer's protocol.

Aliquots (750 ng) of Cy3-labeled cRNA targets were hybridized on Whole Rat Genome Oligo Microarrays (Agilent Technologies). Microarray hybridization and washing were performed using reagents and instruments (hybridization chambers and rotating oven) as indicated by the manufacturer. Microarrays were scanned at 5-*μ*m resolution using a GenePix Personal 4100 A microarray scanner and the GenePix Pro 6.0 acquisition and data-extraction software (Molecular Devices, Sunnyvale, CA, USA). Raw data were processed and analyzed with GeneSpringGX 13 (Agilent Technologies). To remove unreliable data, all genes from all samples were quality-filtered to include only probe data fulfiling all of the following criteria in all replicates of at least one out of four experimental conditions: the spot had <3% of saturated pixels at 532 nm; the spot was not flagged ‘bad’, ‘not found’ or ‘absent’; the spot was detectable well above background (signal-to-noise ratios at 532 nm >10). Filtering data by quality-control criteria short-listed 29 892 probes as our complete data set, out of a total of 41 012 probes present on the microarray. The microarray data have been deposited in the Gene Expression Omnibus (GEO) database under accession no. GSE67788.

Genes in our quality-filtered data set were screened by a one-way ANOVA using Welch's *t*-test, followed by the Benjamini and Hochberg false discovery rate (FDR) procedure as a multiple testing correction and the Tukey’s *post hoc* test. Genes with a corrected *P*-value<0.05 were selected as differentially expressed genes.

### Pathway analysis

To analyze gene expression changes in the context of biological pathways, we used PWEA,^8^ a method to identify functionally related modules of genes, such as KEGG pathways, that correlate with phenotypic differences. PWEA, as implemented in VisANT mining system (http://visant.bu.edu/), differs from other enrichment methods in that it takes account of the functional correlations between genes in the module. This results in higher sensitivity with no loss in specificity.^8^ A PWEA analysis of the four experimental conditions (K25, K5, K5+Igf1 and K5+Pacap) was performed for two pair-wise comparisons (K5+Igf1 and K5+Pacap *versus* K5) using the quality-filtered data set. Overall, 245 *rattus norvegicus* pathways downloaded from KEGG were tested, excluding disease pathways, which are largely uninformative. Statistical significance was estimated from a background distribution generated by 5000 iterations of a permutation test, and the Benjamini–Hochberg FDR procedure using a *P*-value<0.05 as cutoff.

### Real-time quantitative RT-PCR

To validate gene expression profiles observed by microarray technology, deregulated genes following Igf1 and Pacap treatments were re-evaluated using real-time quantitative PCR (RT-PCR) as previously described^80^ (Supplementary Table S2).

## Notes

Supplementary Figure Legends

Supplementary Figure S1A

Supplementary Figure S1B

Supplementary Figure S1C

Supplementary Figure S1D

Supplementary Figure S1E

Supplementary Figure S1F

Supplementary Figure S1G

Supplementary Figure S1H

Supplementary Figure S1I

Supplementary Figure S1L

Supplementary Figure S1M
