# Supplementary material for: Igf1 and Pacap rescue cerebellar granule neurons from apoptosis via a common transcriptional program
Source: Cell Death Discov. 2015 Sep 7;1:15029–. doi: 10.1038/cddiscovery.2015.29 (PMC4773033; doi:10.1038/cddiscovery.2015.29)
Supplement: Supplementary Figure Legends [file cddiscovery201529-s1.doc]

**Figure Legends of Supplementary Materials**

**Figure S1A. Focal adhesion**

Focal adhesions are the connections that form between a cell and its extracellular matrix and are mediated primarily through integrin α/β heterodimeric cell surface receptors. The intracellular tail of integrins interact with numerous signaling molecules, including Fak. The activated Fak forms a complex with Src family kinases, which in turn initiates multiple downstream signaling pathways to regulate cell shape and other different cell functions. Similar morphological alterations are initiated by the binding of growth factors to their respective receptors, emphasizing the considerable crosstalk between adhesion- and growth factor-mediated signaling. (The design of this molecular pathway was based on KEGG Pathway ID “rno04510”).

**Figure S1B. Phagosome**

Phagocytosis plays a central role in defense against infectious agents. The maturation of phagosomes involves interaction with the other membrane organelles, including recycling endosomes, late endosomes and lysosomes. A subset of proteins is involved in the maturation of apoptotic cell-containing phagosomes. Coro1A is part of the phagocytic cup. Fc gamma RII alpha contains the immunoreceptor tyrosine-based activation motif (ITAM) within its cytoplasmic tails or in associated subunits. Dync1li2 is a member of the dynein multi-complex protein which interacts with members of the Tubulin family, causing the maturation of sorting endosomes into late endosomes. CD-MPR is part of the lysosome. (The design of this molecular pathway was based on KEGG Pathway ID “rno04145”).

**Figure S1C. Antigen Processing and Presentation**

The process by which antigen-presenting cells digest proteins from inside or outside the cell helps to detect signs of infection or abnormal cell growth. In rescued CGNs, this pathway involves a range of molecules that are located in transmembrane, cytoplasmic and nuclear compartments. (The design of this molecular pathway was based on KEGG Pathway ID “rno04612”).

**Figure S1D. Metabolism**

Cellular metabolism involves complex sequences of controlled biochemical reactions, better known as metabolic pathway. The main chemical reactions of CGN metabolism are organized into four subgroups: Glycine, serine and threonine metabolism (KEGG Pathway ID: rno00260), Glutathione metabolism (KEGG Pathway ID: rno00480) and Sfingolipid metabolism. The design of this molecular pathway was based on KEGG Pathway ID “rno00600”.

**Figure S1E. Chemokine signaling**

The chemokine signal is transduced by G-protein coupled receptors, which in turn activate diverse downstream pathways, including AKT, PKA and Calcium signaling. (The design of this molecular pathway was based on KEGG Pathway ID “rno04062”).

**Figure S1F. Neuroactive ligand-receptor interaction**

Interaction of a variety of signaling molecules is crucial to mediate neuronal functions. Pthr1 is a GPCR that once bound to the Parathyroid hormone (Pth) activates the cAMP-dependent protein kinase (PKA) pathway. Gal, OTR, F2rl3i bind G protein-coupled receptors including GalR2 and then activates PLC signaling. Adenosine A1 receptor receptor modulates glutamate secretion through G-protein signaling that is dependent on adenosine concentration. P2X6 initiates the via purinergic signaling. (The design of this molecular pathway was based on KEGG Pathway ID “rno04080”).

**Figure S1G. Olfactory transduction**

Odorant binding to olfactory receptors causes G-protein trimeric complex dissociation, resulting in the release of activated G-protein alpha-olf and G-protein beta1/gamma13 subunits. G-protein alpha-olf activates adenylate cyclase type III that in turn catalyzes cyclic AMP formation. Cyclic AMP binds to and opens olfactory cyclic nucleotide gated (CNG) channels, which allow Ca2+ to flow into the cell. An increase in intracellular calcium causes calcium-gated chloride channel activation. Signal termination in olfactory sensory neurons is achieved through the Ca2+/Calmodulin pathway. (The design of this molecular pathway was based on KEGG Pathway ID “rno04740”).

**Figure S1H. Calcium signaling**

In neurons, calcium signaling is the major second messenger involved in a variety of intracellular pathways. Cells use external source of Ca2+ signal by activating entry channels with widely different properties. The other principal source of Ca2+ for signalling is the internal stores that are located primarily in the endoplasmic/sarcoplasmic reticulum, in which inositol-1,4,5-trisphosphate receptors (IP3Rs) or ryanodine receptors (RYRs) regulate the release of Ca2+ into the cytosol. Various molecules, such as oxytocin, also control the release of Ca2+, stimulates an increased intracellular calcium. High levels of intracelluar Ca('2+) lead to mitochondrial Ca('2+) overloads, induce mitochondrial membrane depolarization and stimulate Ca('2+) efflux from the mitochondria (The design of this molecular pathway was based on KEGG Pathway ID “rno04020”).

**Figure S1I. GABAergic Synapse**

Gamma-Aminobutyric acid (GABA) is the most abundant inhibitory neurotransmitter in the mammalian brain that in GABAergic terminals is formed from L-Glutamic acid in an enzymatic reaction mediated by GAD1 and GAD2. GABA binds to different GABAergic receptors, such as such as GABA-A and GABA-B. GABA-A receptors is ionotropic and mediates fast GABA responses by triggering chloride channel openings, while GABA-B receptors is metabotropic and mediates slower GABA responses by activating G-proteins and influencing second messenger systems. (The design of this molecular pathway was based on KEGG Pathway ID “rno04727”).

**Figure S1L. Hedgehog and TGF-beta signaling**

Hedgehog-family ligands regulate the transcription of target genes, such as Ptc, in a dose-responsive manner. This in turn activates β-arrestin, Smoothened and Gli target genes, resulting in well-coordinated transcriptional events (KEGG Pathway ID: rno04340). This cascade integrates signals from WNT and ii) TGF-β routes. The Wnt ligands bind to Frizzled receptors that induce the activation of Dvl. The transcriptional effects of Wnt ligand are mediated via Rac1-dependent nuclear translocation of β-catenin and the subsequent recruitment of Tcf/Lef DNA-binding factors as co-activators for transcription. The TGF-β superfamily signaling is initiated with ligand-induced oligomerization of serine/threonine receptor kinases and phosphorylation of the Smad2 and Smad3. The expression of inhibitory Smad 6 is induced by a negative feedback loop (The design of this molecular pathway was based on KEGG Pathway ID “rno04350”).

**Figure S1M. MAPK signaling**

The MAPK signaling cascade is activated by a wide variety of receptors, including receptor tyrosine kinases and ion channels. The architecture of the pathway includes a set of adaptors (Shc, GRB2, etc.) linking the receptors to a guanine nucleotide exchange factors (SOS, etc.) that transduce the signal to small GTP-binding proteins (i.e. Ras) and in turn activate MAPKKK (Raf), MAPKK (MEK1/2) and MAPK (Erk). An activated Erk dimer phosphorylates a variety of transcription factors (The design of this molecular pathway was based on KEGG Pathway ID “rno04010”).

**Figure S2. Explanation of the symbols used in GeneGo Metacore pathway map**

The figure legend shows the set of symbols whereby network objects and interactions between objects are indicated in the figure 2. Downward thermometers have a blue color and indicate down-regulated expression, whereas upward thermometers have a red color and indicate up-regulated expression of genes associated with CGNs rescue by Igf1 and Pacap treatments.
