# Supplementary figures and images for: Igf1 and Pacap rescue cerebellar granule neurons from apoptosis via a common transcriptional program
Source: Cell Death Discov. 2015 Sep 7;1:15029–. doi: 10.1038/cddiscovery.2015.29 (PMC4773033; doi:10.1038/cddiscovery.2015.29)

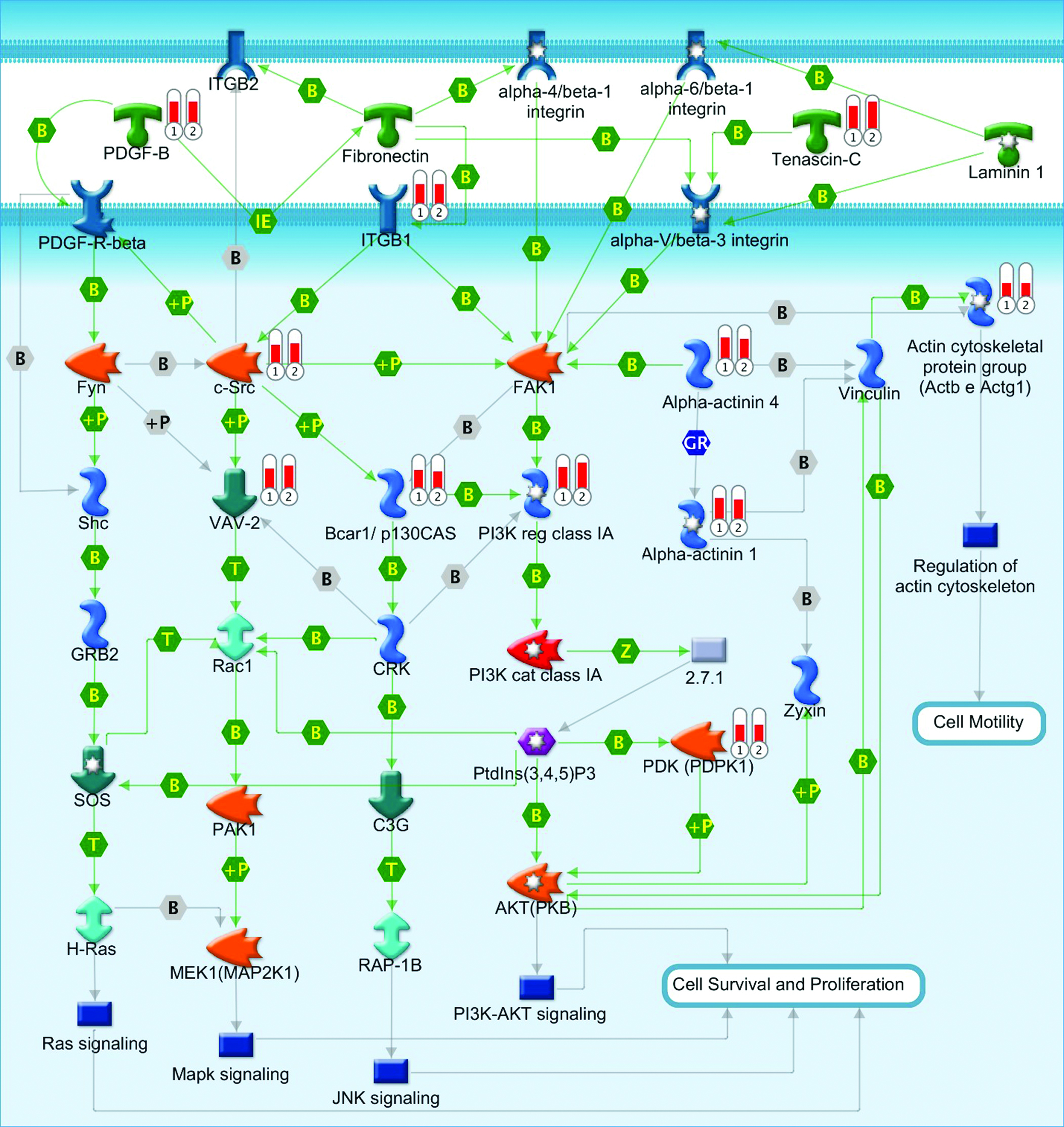

Supplement: Supplementary Figure S1A [file cddiscovery201529-s2.jpg]

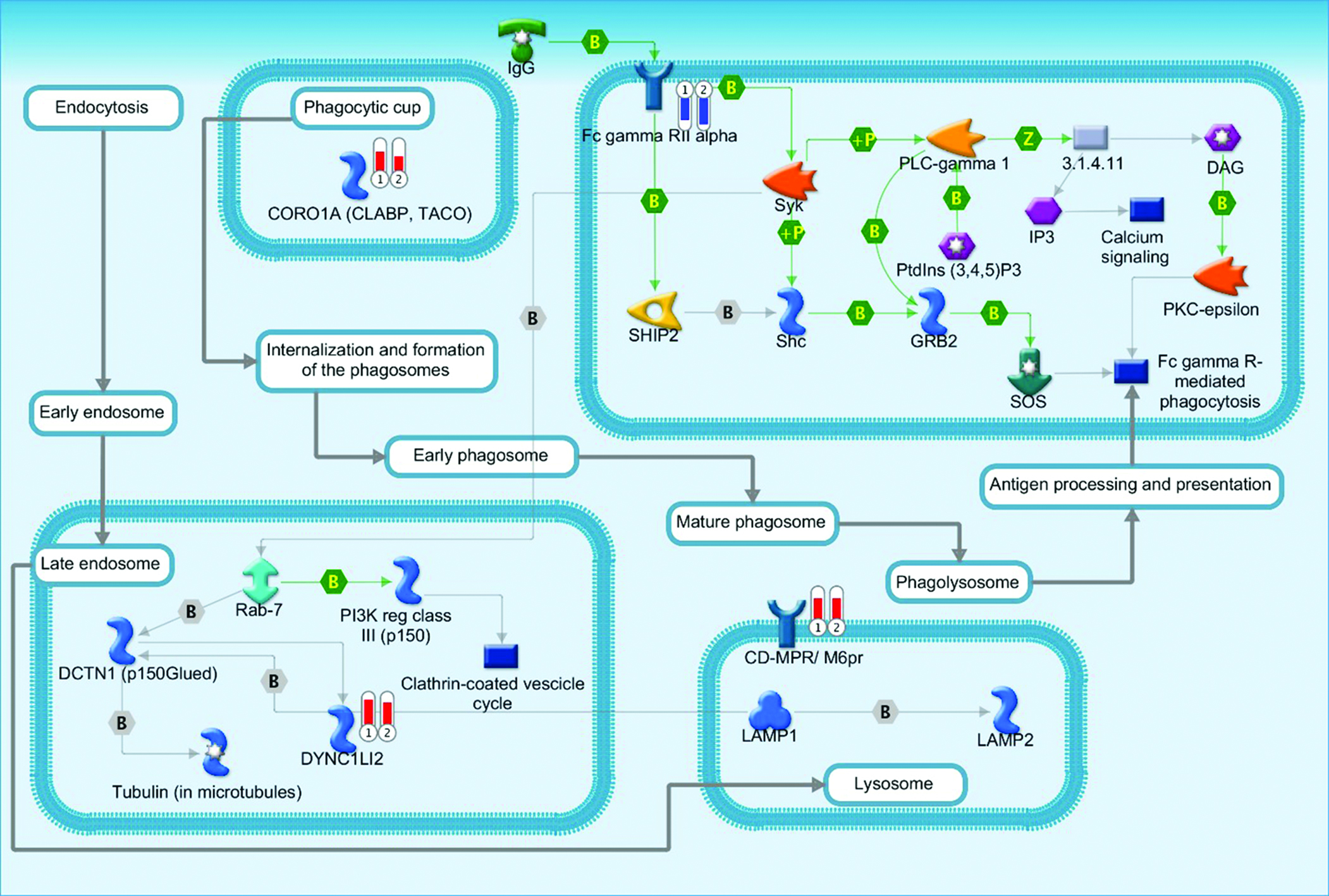

Supplement: Supplementary Figure S1B [file cddiscovery201529-s3.jpg]

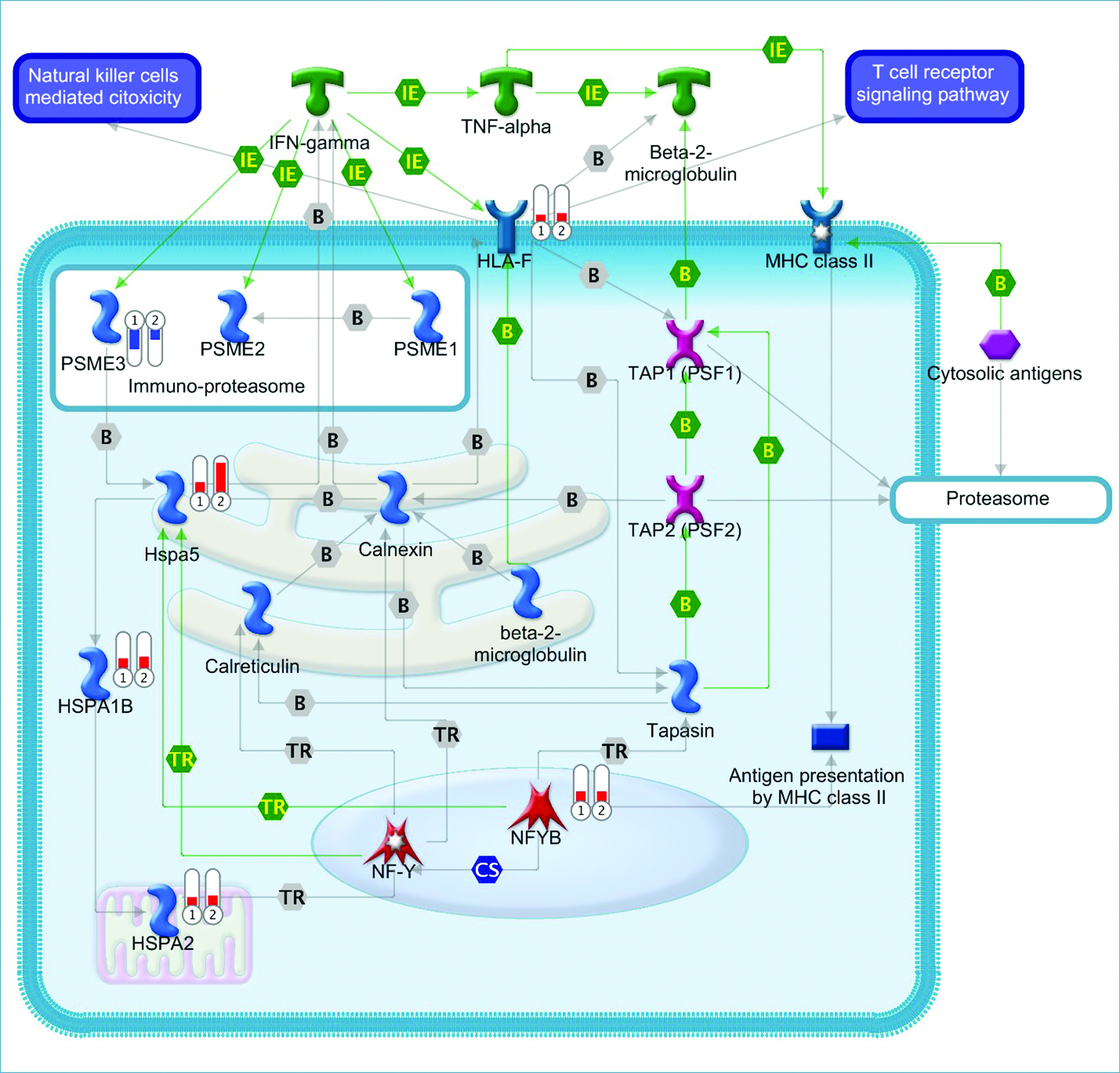

Supplement: Supplementary Figure S1C [file cddiscovery201529-s4.jpg]

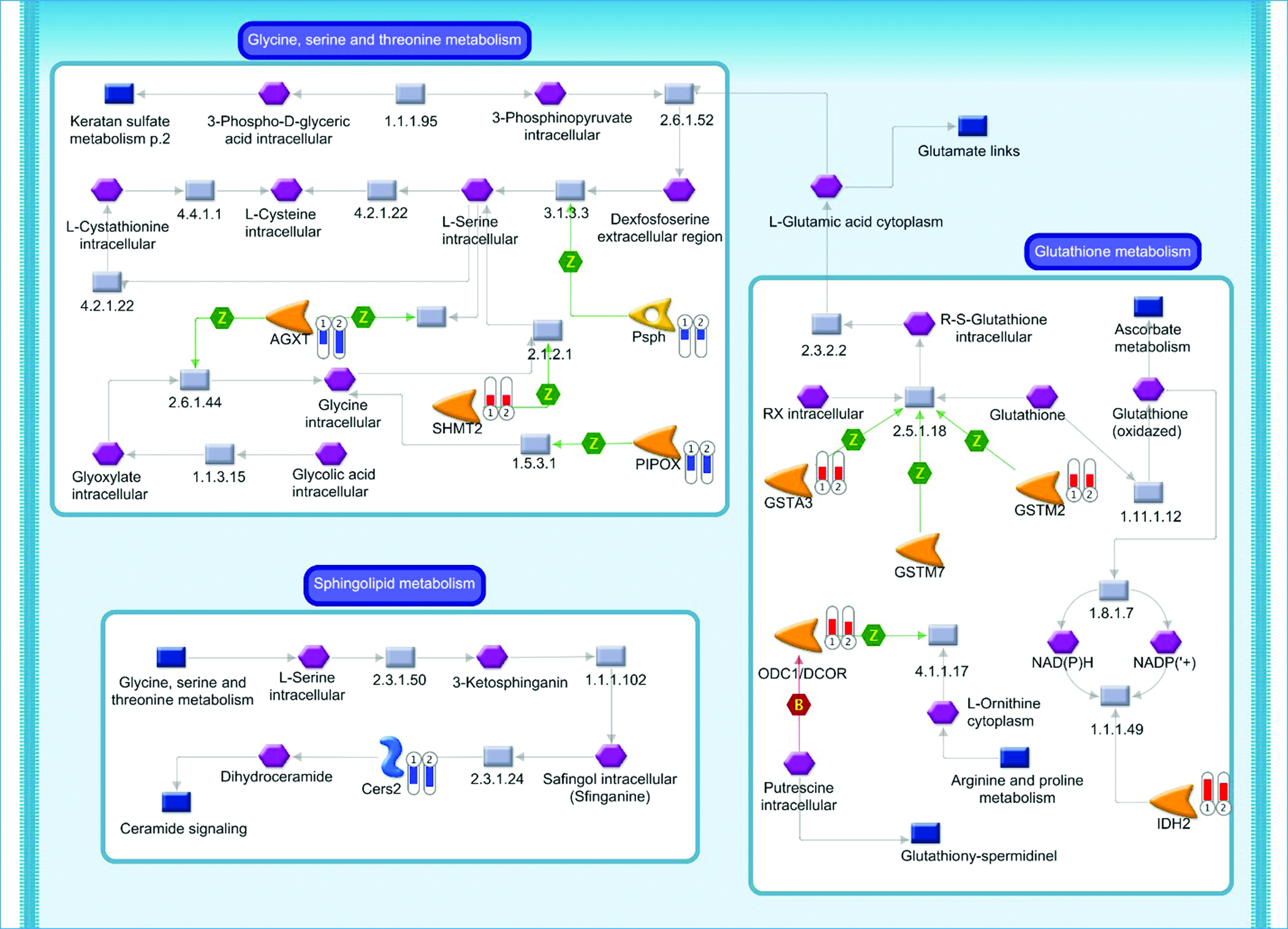

Supplement: Supplementary Figure S1D [file cddiscovery201529-s5.jpg]

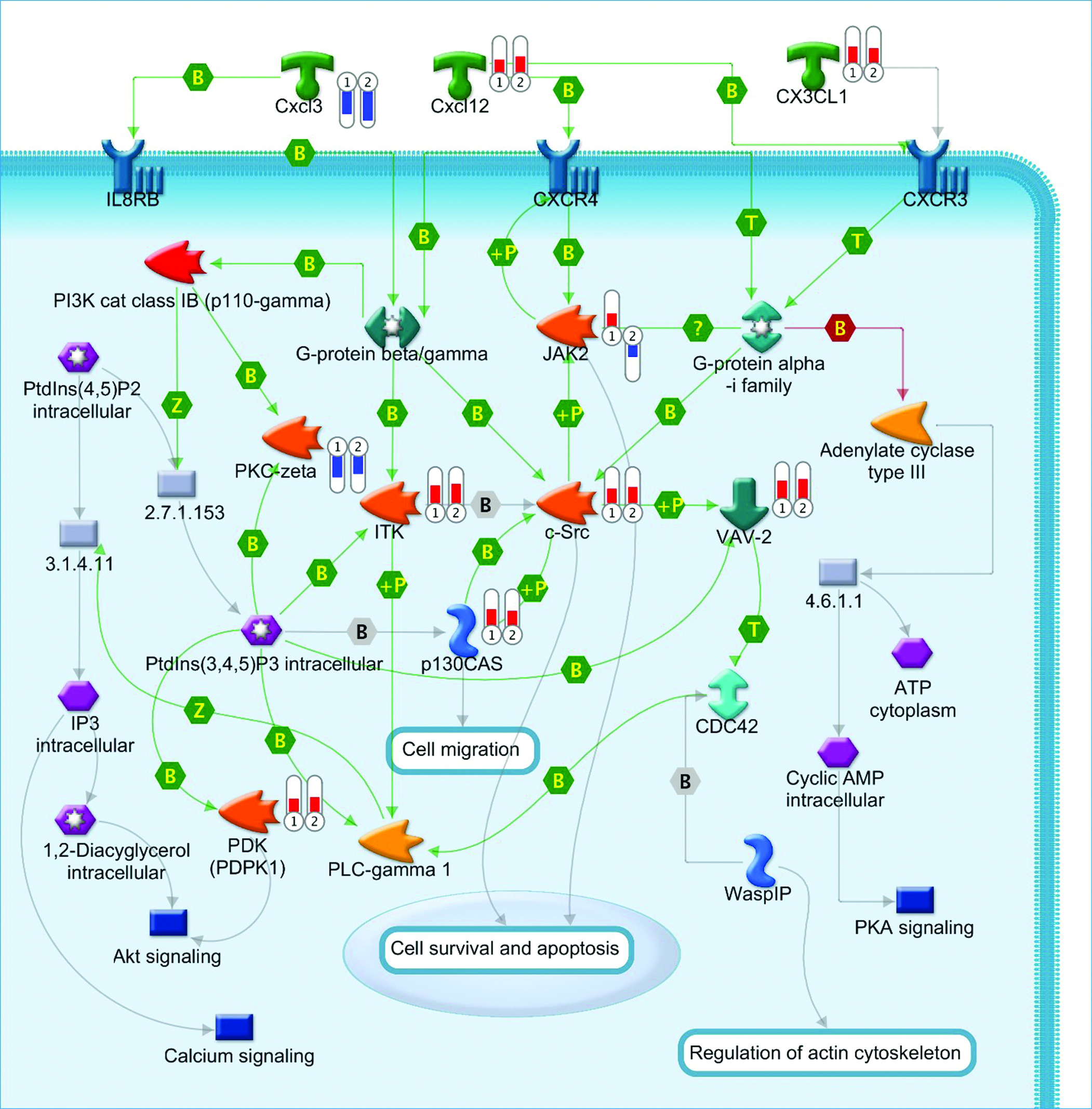

Supplement: Supplementary Figure S1E [file cddiscovery201529-s6.jpg]

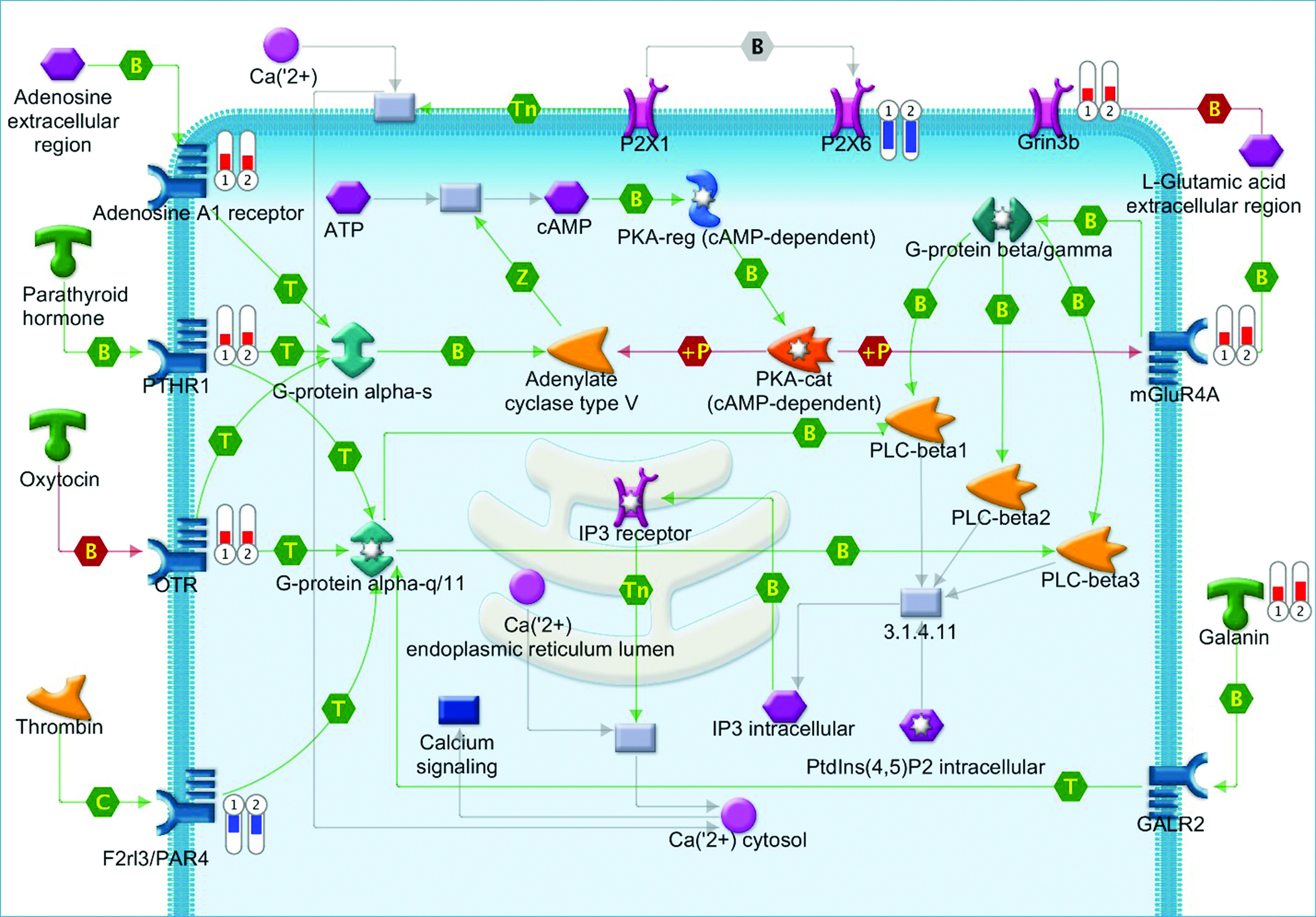

Supplement: Supplementary Figure S1F [file cddiscovery201529-s7.jpg]

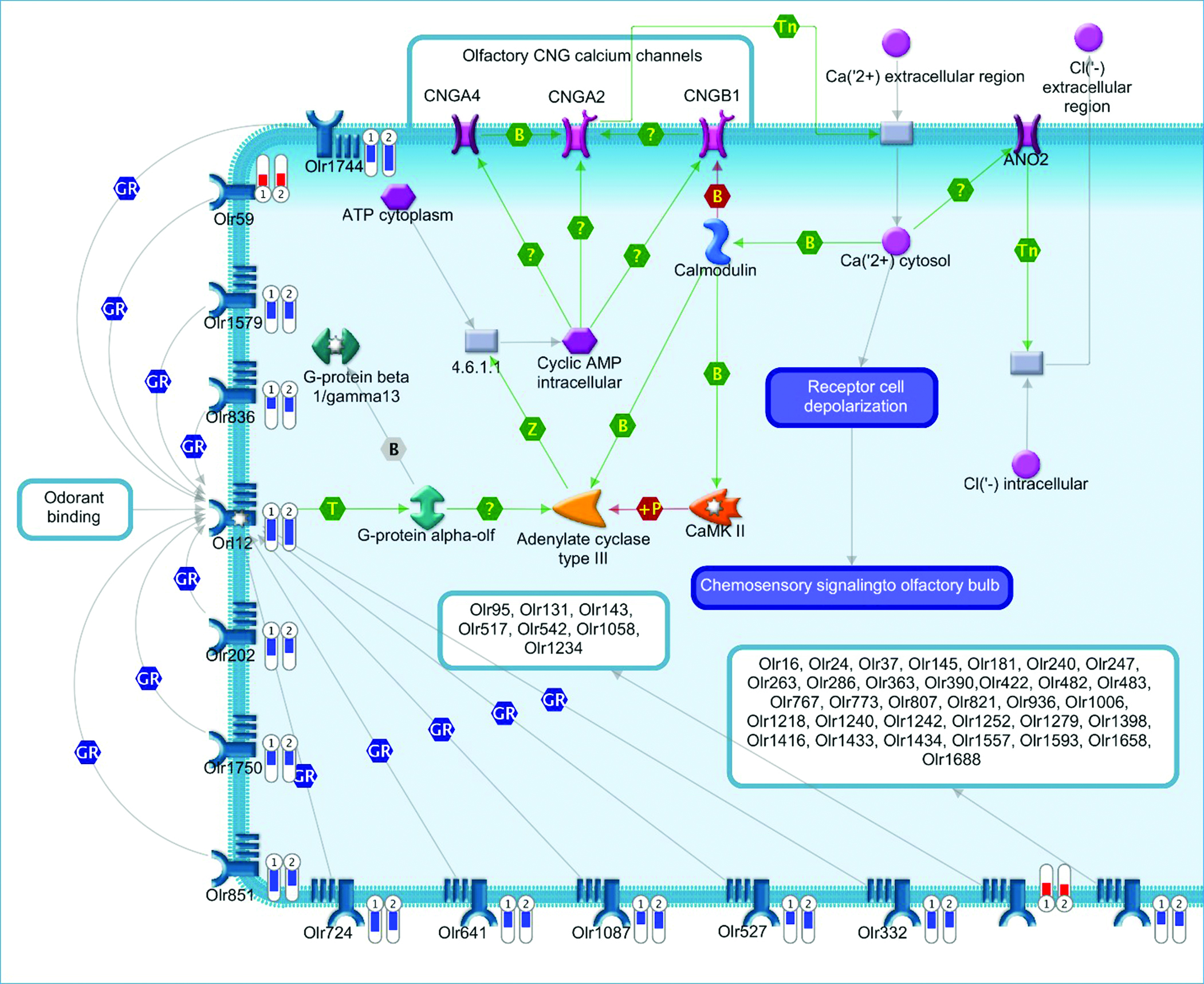

Supplement: Supplementary Figure S1G [file cddiscovery201529-s8.jpg]

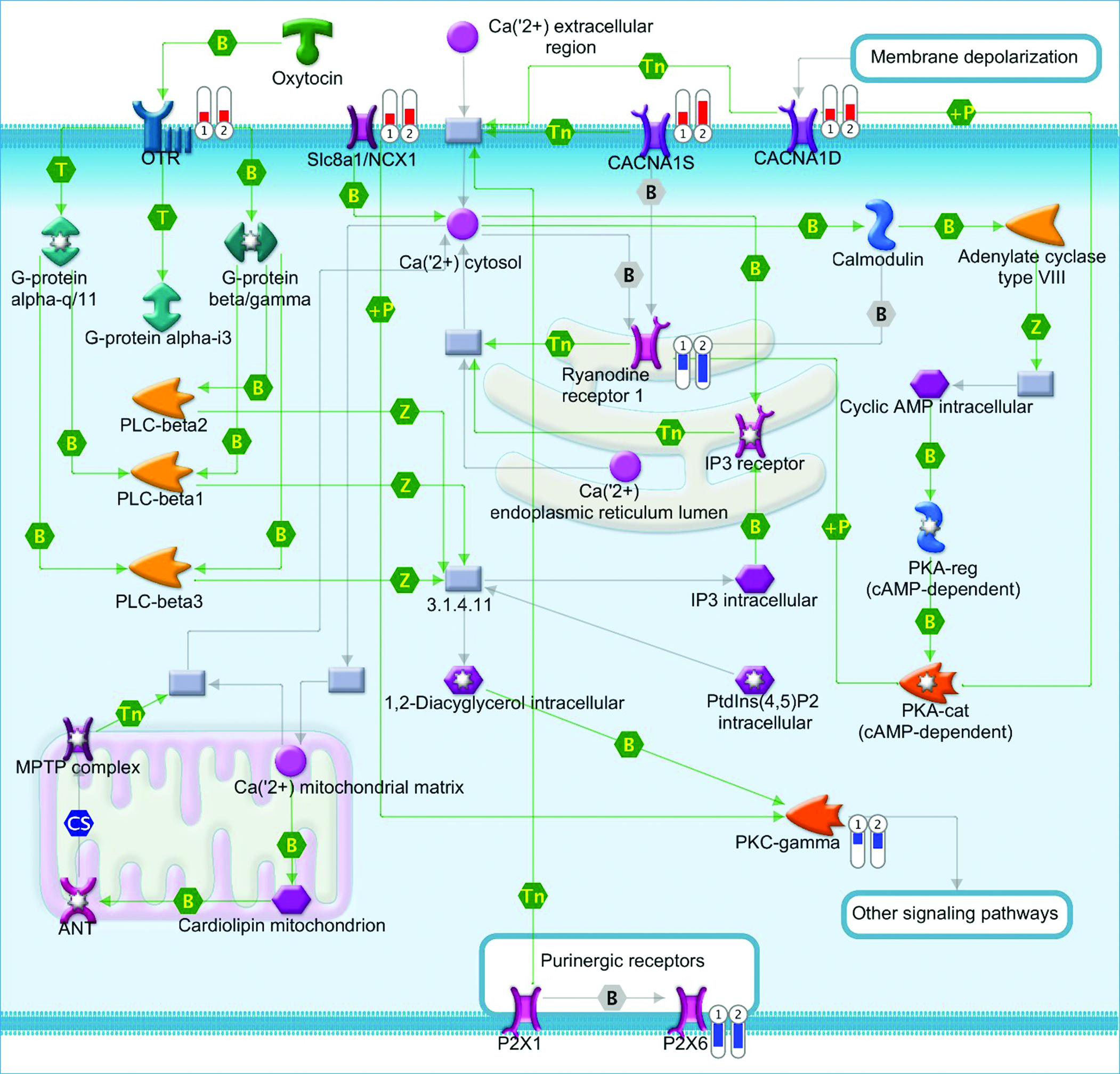

Supplement: Supplementary Figure S1H [file cddiscovery201529-s9.jpg]

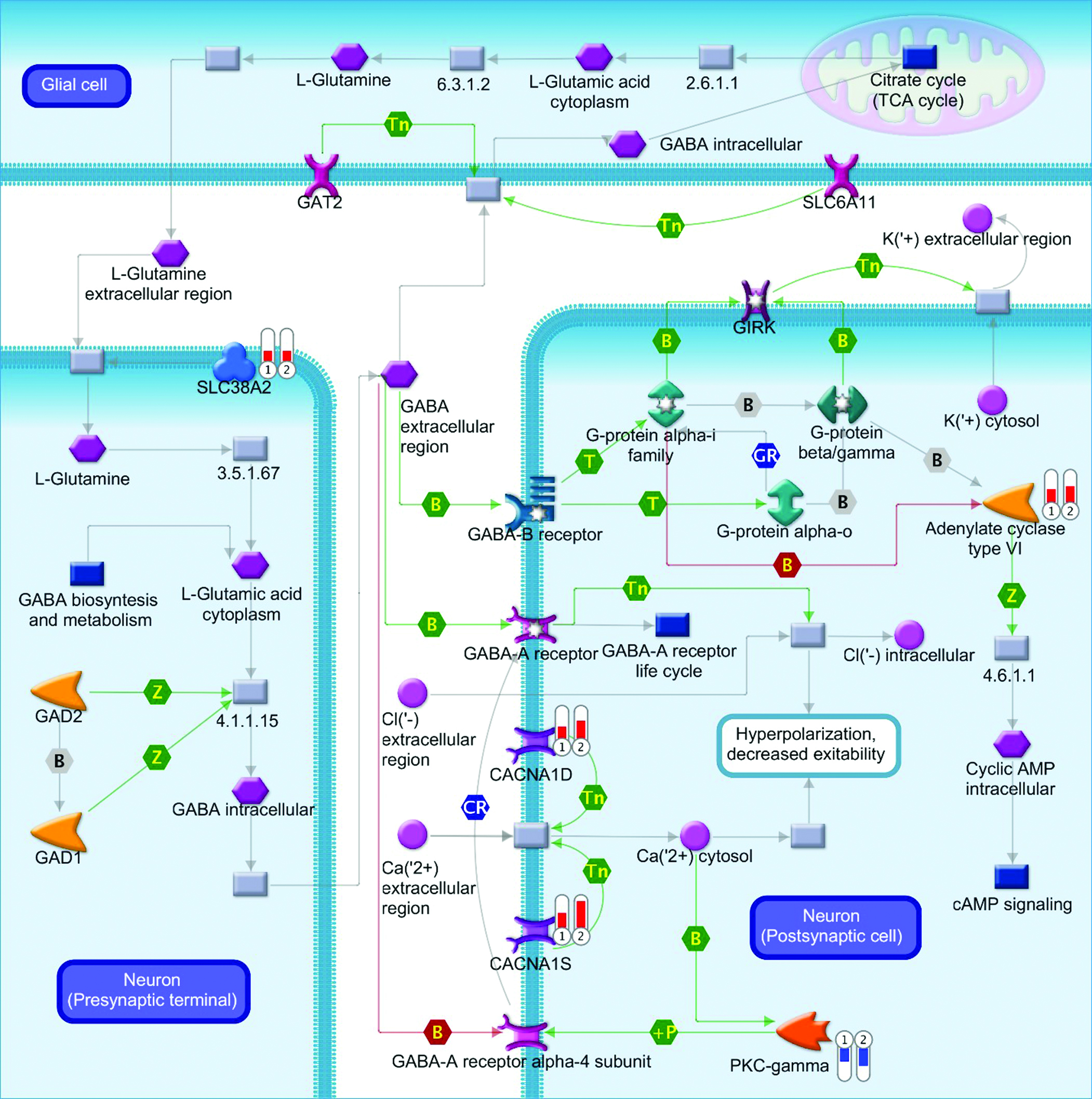

Supplement: Supplementary Figure S1I [file cddiscovery201529-s10.jpg]

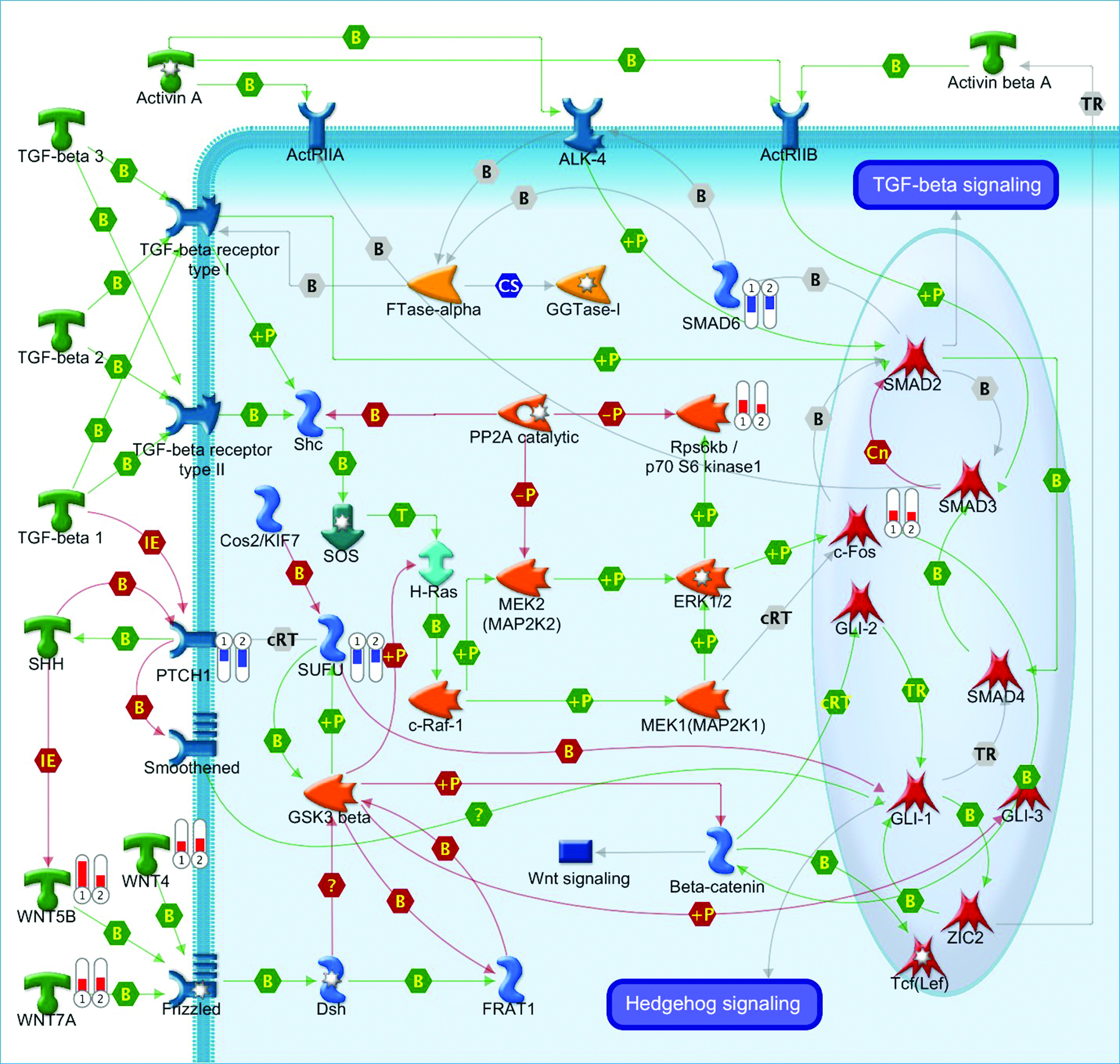

Supplement: Supplementary Figure S1L [file cddiscovery201529-s11.jpg]

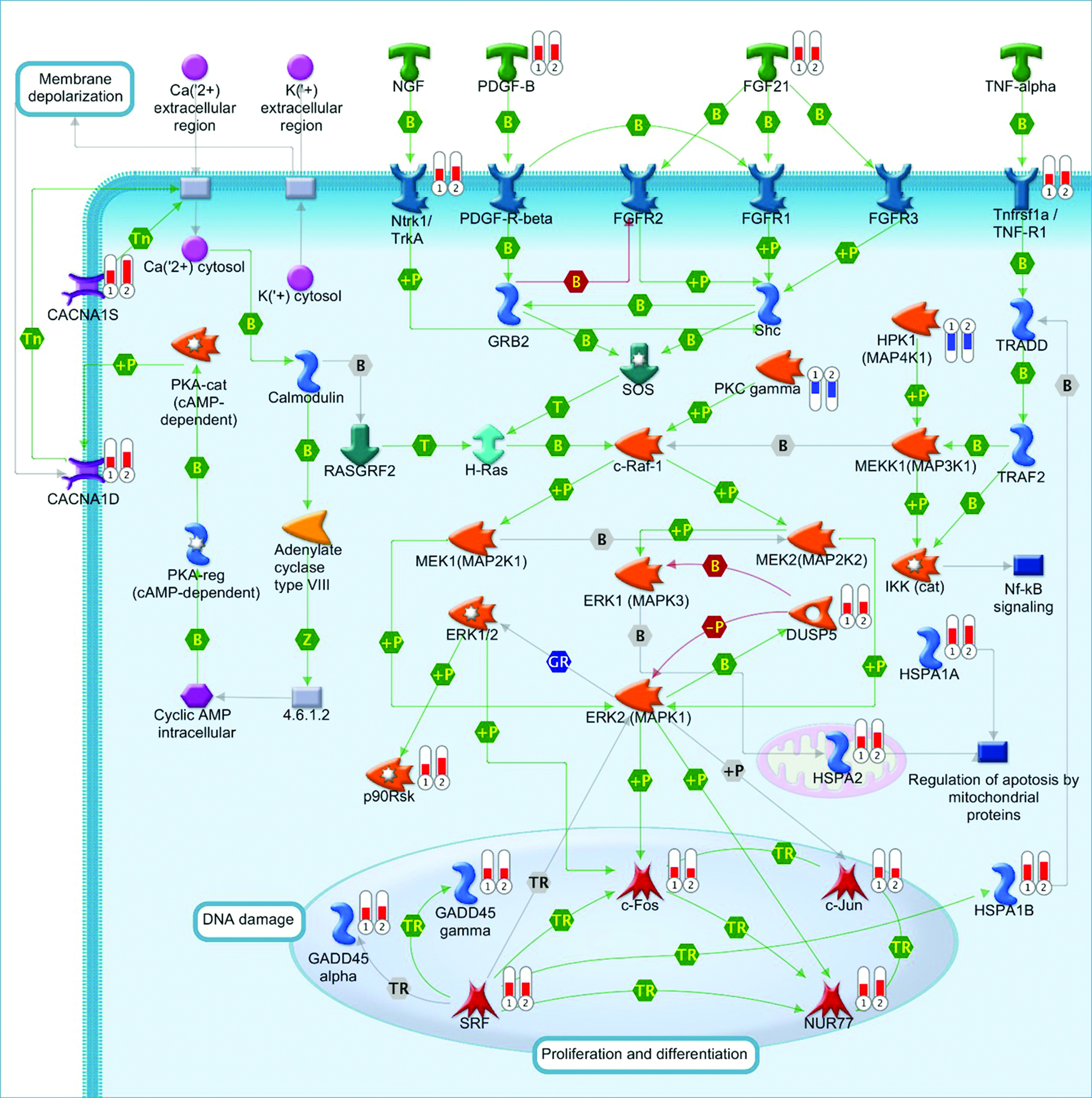

Supplement: Supplementary Figure S1M [file cddiscovery201529-s12.jpg]

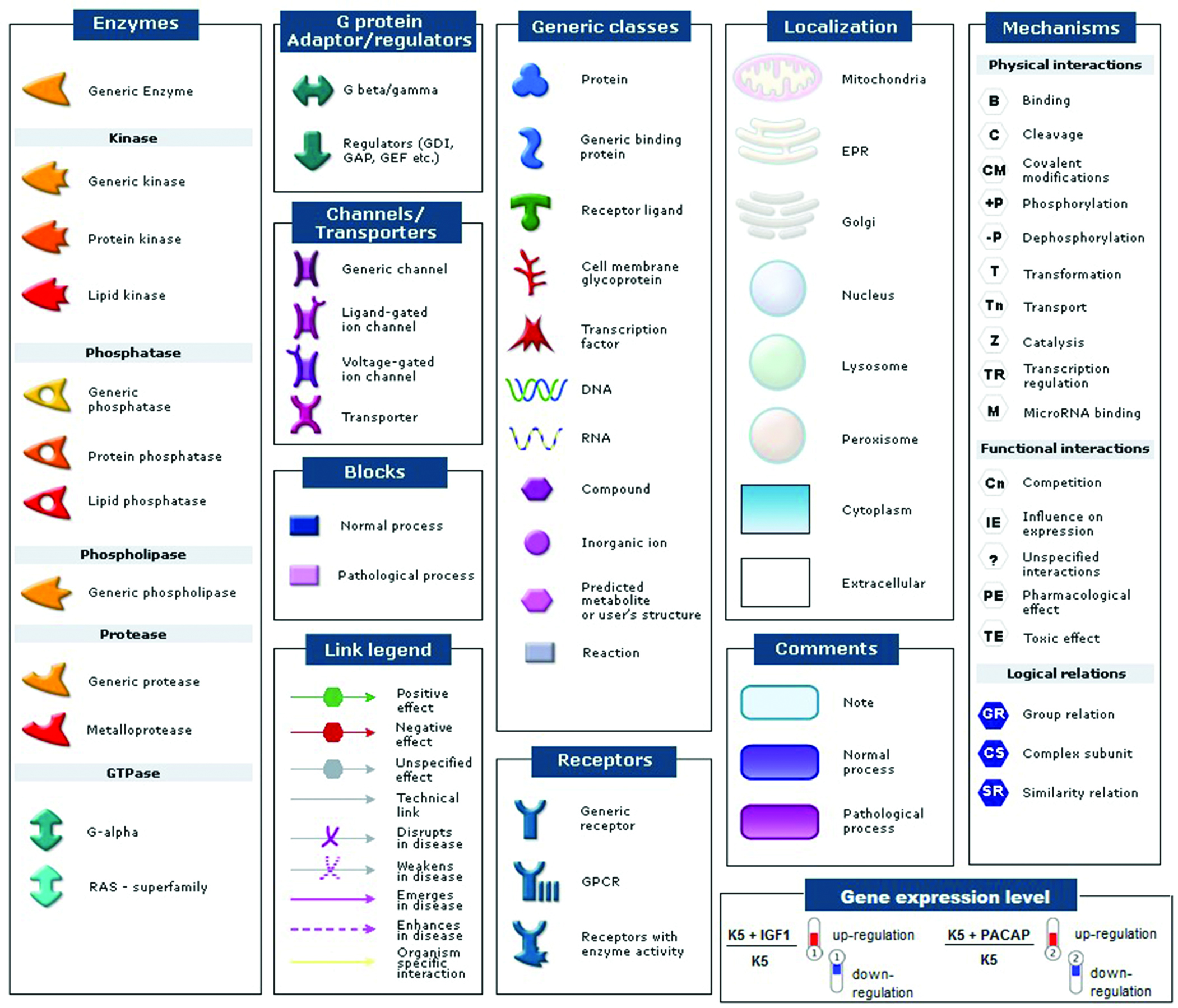

Supplement: Supplementary Figure S2 [file cddiscovery201529-s13.jpg]
