# Supplementary material for: Igf1 and Pacap rescue cerebellar granule neurons from apoptosis via a common transcriptional program
Source: Cell Death Discov. 2015 Sep 7;1:15029–. doi: 10.1038/cddiscovery.2015.29 (PMC4773033; doi:10.1038/cddiscovery.2015.29)
Supplement: Supplementary Table S1 [file cddiscovery201529-s14.doc]

**Table S1.** Potential drug targets at the intersection of neuronal apoptosis and survival and compounds inferred by GeneGo Metacore software

| **#** | **Gene Symbol** | **Target** | **Drug** | **Effect** |
| --- | --- | --- | --- | --- |
| 1. | Ntrk1 | TrkA | Lestaurtinib extracellular region | Inhibition |
| 2. | Odc1 | DCOR | Eflornithine intracellular | Inhibition |
|  |  |  | N(1),N(11)-Diethylnorspermine intracellular | Inhibition |
| 3. | Ryr1 | Ryanodine receptor 1 | Dantrolene intracellular | Inhibition |
| 4. | Pdgfb | PDGF-B | Suramin extracellular region | Inhibition |
| 5. | Cacna1s | CACNA1S | MEM1003 extracellular region | Inhibition |
|  |  |  | Dronedarone extracellular region | Inhibition |
|  |  |  | Clevidipine extracellular region | Inhibition |
| 6. | Grin3b | NR3B | Memantine extracellular region | Inhibition |
|  |  |  | Ketamine extracellular region | Inhibition |
| 7. | Rps6kb1 | p70 S6 kinase1 | XL418 intracellular | Inhibition |
| 8. | Pdk1 | PDK1 | Dichloroacetic acid intracellular | Inhibition |
| 9. | Adora1 | Adenosine A1 receptor | Caffeine extracellular region | Inhibition |
|  |  |  | Naxifylline extracellular region | Inhibition |
|  |  |  | Rolofylline extracellular region | Inhibition |
|  |  |  | Propentofylline extracellular region | Activation |
|  |  |  | Selodenoson extracellular region | Activation |
|  |  |  | Adenosine extracellular region | Activation |
|  |  |  | Tecadenoson extracellular region | Activation |
|  |  |  | Tonapofylline extracellular region | Inhibition |
| 10. | Src | c-Src | Dasatinib intracellular | Inhibition |
|  |  |  | Nintedanib intracellular | Inhibition |
|  |  |  | Saracatinib intracellular | Inhibition |
|  |  |  | Cediranib intracellular | Inhibition |
| 11. | Cacna1d | CACNA1D | Clevidipine extracellular region | Inhibition |
|  |  |  | Isradipine extracellular region | Inhibition |
|  |  |  | Dronedarone extracellular region | Inhibition |
|  |  |  | MEM1003 extracellular region | Inhibition |
| 12. | Cdh2 | N-cadherin | ADH-1 intracellular | Inhibition |
| 13. | Tgm2 | TGM2 | Cysteamine intracellular | Inhibition |
| 14. | Atp1a1 | ATP1A1 | Trichlormethiazide intracellular | Inhibition |
| 15. | Casp7 | Caspase-7 | As(,2)O(,3) intracellular | Activation |
|  |  |  | z-vad-FMK intracellular | Inhibition |
| 16. | Nr1h4 | FXR | Apomine intracellular | Activation |
| 17. | Ret | RET | Sunitinib extracellular region | Inhibition |
|  |  |  | Cabozantinib extracellular region | Inhibition |
|  |  |  | Sorafenib intracellular | Inhibition |
| 18. | Crhr1 | CRHR1 | NBI37582 extracellular region | Inhibition |
|  |  |  | NBI34041 extracellular region | Inhibition |
| 19. | Fntb | FTase-beta | L-778,123 intracellular | Inhibition |
|  |  |  | Tipifarnib intracellular | Unspecified |
|  |  |  | BMS214662 intracellular | Inhibition |
| 20. | Car4 | Carbonic anhydrase IV | Acetazolamide intracellular | Inhibition |
|  |  |  | Celecoxib intracellular | Inhibition |
|  |  |  | Hydroflumethiazide intracellular | Inhibition |
|  |  |  | Indisulam intracellular | Inhibition |
|  |  |  | Trichlormethiazide intracellular | Inhibition |
| 21. | Nae1 | APP-BP1 | Pevonedistat intracellular | Inhibition |
| 22. | Atp12a | ATP12A | Rabeprazole intracellular | Inhibition |
| 23. | Gart | PUR2 | Pelitrexol intracellular | Inhibition |
| 24. | Tuba4a | Tubulin alpha-4° | Entasobulin intracellular | Inhibition |
| 25. | Gaa | GAA | Miglustat intracellular | Inhibition |
|  |  |  | Voglibose intracellular | Inhibition |
| 26. | Slc6a2 | NET | Venlafaxine extracellular region | Inhibition |
|  |  |  | Duloxetine extracellular region | Inhibition |
|  |  |  | Nefazodone extracellular region | Inhibition |
|  |  |  | Atomoxetine extracellular region | Inhibition |
|  |  |  | Bupropion extracellular region | Inhibition |
|  |  |  | Clomipramine extracellular region | Inhibition |
|  |  |  | Bicifadine extracellular region | Inhibition |
|  |  |  | DOV216303 extracellular region | Inhibition |
|  |  |  | Maprotiline extracellular region | Inhibition |
|  |  |  | Sibutramine extracellular region | Inhibition |
|  |  |  | Betanidine intracellular | Unspecified |
|  |  |  | Guanadrel extracellular region | Inhibition |
|  |  |  | Amoxapine extracellular region | Inhibition |
|  |  |  | Amineptine extracellular region | Inhibition |
|  |  |  | Phentermine extracellular region | Inhibition |
|  |  |  | Dexmethylphenidate extracellular region | Inhibition |
| 27. | Kcna6 | KV1.6 | Dalfampridine extracellular region | Inhibition |
| 28. | Csf1r | M-CSF receptor | Sunitinib extracellular region | Inhibition |
|  |  |  | Cediranib intracellular | Inhibition |
| 29. | Mmp16 | MMP-16 | Batimastat extracellular region | Inhibition |
| 30. | Txnrd1 | TXNRD1 | Evofosfamide intracellular | Inhibition |
|  |  |  | Motexafin gadolinium intracellular | Inhibition |
| 31. | Hdac7 | HDAC7 | Vorinostat intracellular | Inhibition |
|  |  |  | Belinostat intracellular | Inhibition |
| 32. | Gabrb2 | GABA-A receptor beta-2 subunit | Clomethiazole extracellular region | Activation |
| 33. | Kcnk2 | KCNK2 | Dofetilide extracellular region | Inhibition |
|  |  |  | Sipatrigine extracellular region | Inhibition |
| 34. | Kcnc1 | Kv3.1 | Dalfampridine extracellular region | Inhibition |
| 35. | Psenen | Pen-2 | Begacestat extracellular region | Inhibition |
| 36. | Pim2 | Pim-2 | SGI-1776 intracellular | Inhibition |
| 37. | Top2a | TOP2 alpha | NK109 intracellular | Inhibition |
|  |  |  | Ciprofloxacin intracellular | Inhibition |
|  |  |  | NSC601316 intracellular | Inhibition |
|  |  |  | Piroxantrone intracellular | Inhibition |
|  |  |  | Etoposide intracellular | Inhibition |
|  |  |  | Entasobulin intracellular | Inhibition |
|  |  |  | Aclarubicin intracellular | Inhibition |
|  |  |  | Annamycin intracellular | Inhibition |
|  |  |  | Mitoxantrone intracellular | Inhibition |
|  |  |  | Losoxantrone intracellular | Inhibition |
|  |  |  | Teniposide intracellular | Inhibition |
|  |  |  | GL331 intracellular | Inhibition |
|  |  |  | TOP53 intracellular | Inhibition |
|  |  |  | Epirubicin intracellular | Inhibition |
|  |  |  | Amsacrine intracellular | Inhibition |
|  |  |  | Intoplicine intracellular | Inhibition |
|  |  |  | Elsamitrucin intracellular | Inhibition |
| 38. | Pde4b | PDE4B | Pentoxifylline intracellular | Inhibition |
|  |  |  | Ibudilast intracellular | Inhibition |
|  |  |  | Roflumilast intracellular | Inhibition |
|  |  |  | Theophylline intracellular | Inhibition |
|  |  |  | Papaverine intracellular | Inhibition |
|  |  |  | MK-0359 intracellular | Inhibition |
| 39. | Cdk4 | CDK4 | Palbociclib intracellular | Inhibition |
|  |  |  | Riviciclib intracellular | Inhibition |
|  |  |  | Alvocidib intracellular | Inhibition |
|  |  |  | AT7519M intracellular | Inhibition |
|  |  |  | Roniciclib intracellular | Inhibition |
|  |  |  | R547 intracellular | Inhibition |
| 40. | Hdac4 | HDAC4 | Belinostat intracellular | Inhibition |
|  |  |  | Vorinostat intracellular | Inhibition |
| 41. | Ret | RET | Sunitinib extracellular region | Inhibition |
|  |  |  | Cabozantinib extracellular region | Inhibition |
|  |  |  | Sorafenib intracellular | Inhibition |
| 42. | Pdpk1 | PDK (PDPK1) | Celecoxib intracellular | Inhibition |
| 43. | Timp2 | TIMP2 | Pravastatin extracellular region | Inhibition |
| 44. | Flt3 | FLT3 | Sunitinib extracellular region | Inhibition |
|  |  |  | Lestaurtinib extracellular region | Inhibition |
|  |  |  | Sorafenib intracellular | Inhibition |
|  |  |  | Dovitinib extracellular region | Inhibition |
|  |  |  | Tandutinib extracellular region | Inhibition |
|  |  |  | Cabozantinib extracellular region | Inhibition |
|  |  |  | Linifanib extracellular region | Inhibition |
