# Supplementary material for: Igf1 and Pacap rescue cerebellar granule neurons from apoptosis via a common transcriptional program
Source: Cell Death Discov. 2015 Sep 7;1:15029–. doi: 10.1038/cddiscovery.2015.29 (PMC4773033; doi:10.1038/cddiscovery.2015.29)
Supplement: Supplementary Table S2 [file cddiscovery201529-s15.doc]

**Table S2.** Validation of microarray data by real-time quantitative RT-PCR.
